# Self-compassion and sleep quality: Examining the mediating role of taking a proactive health focus and cognitive emotional regulation strategies

**DOI:** 10.1177/13591053211047148

**Published:** 2021-09-20

**Authors:** Brittany N Semenchuk, Samantha Onchulenko, Shaelyn M Strachan

**Affiliations:** 1 University of Manitoba, Canada; 2 University of North Dakota School of Law, USA

**Keywords:** common humanity, health, mindfulness, self-kindness, self-regulation

## Abstract

Sleep quality (SQ) impacts health yet many university students get poor sleep. Self-compassion (SC)—care for oneself during challenging times—is associated with SQ yet *how* SC has these effects is unclear. This study cross-sectionally examined whether SC is negatively related to poor SQ and whether proactive health focus and cognitive emotional regulation strategies (CERS) mediate this relationship. University students (*N* = 193) self-reported SC, proactive health focus, CERS, and SQ. SC negatively associated with poor SQ (*r* = −0.34) and self-blame mediated this (*b* = 0.08, 95% CI [0.01, 0.16]). SC may improve SQ through reducing self-blame.

Sleep influences mental, emotional and physical health, and quality of life (Mukherjee et al., 2015). Those with insufficient sleep have higher risks of cardiovascular disease, diabetes (Buxton and Marcelli, 2010), and anxiety (Roberts and Duong, 2017). Despite the widespread health implications of sleep, people report poor sleep quality, and sleep difficulties (Puterbaugh, 2011). For adults 18–64 years of age, one-third report getting less than the recommended 7–9 hours of sleep per night and 43% of men and 55% of women report having trouble going to sleep or staying asleep for the majority of time (Statistics Canada, 2017).

University students often fail to get sufficient quality sleep. Indeed, fewer than 1/3 of this population get the recommended 8 hours of sleep per night and 1/4 report getting less than 6.5 hours of sleep per night (Lund et al., 2010), according to self-reports. These short sleep durations have been linked to diminished cognitive functioning, reduced productivity (Taylor and Bramoweth, 2010), and increased risk of mental health disorders among university students (Dickinson et al., 2018).

## Factors influencing sleep quality

Many factors, including academic, employment, and social demands increase the risk of poor sleep quality among university students (Coveney, 2013; Foulkes et al., 2019). When a student perceives they are unable to cope with these competing demands a stress response is evoked (Lazarus and Launier, 1978; Lovallo, 2015), which may take the form of challenging emotions such as self-blame (Ding and Curtis, 2020) for events such as poor academic performance. Prolonged stress can lead to anxiety (Miklósi et al., 2014) and depression (Hammen, 2005) and ultimately, poor sleep quality (Wang and Bíró, 2021). The capability to cope with emotionally challenging situations (such as demands from university) by using adaptive cognitive emotional regulation is imperative (Langer et al., 2020). Adaptive cognitive emotional regulation strategies (e.g. positive reappraisal) are negatively associated with stress (Amaral et al., 2015) and have been found to positively impact sleep quality (Gellis et al., 2013; Palmer et al., 2018). Among adolescents (*N* = 10,148), Palmer et al. (2018) found that using problem-solving strategies to cope with unpleasant emotions is negatively associated with poor sleep quality. Maladaptive strategies (e.g. self-blame) are positively associated with stress (Amaral et al., 2015) and negatively impact sleep quality among university students (Amaral et al., 2016, 2018). These maladaptive cognitive emotional regulation strategies (e.g. self-blame) mediate the relationship between stress and sleep quality among students (Yan et al., 2018). Importantly, interventions designed to improve cognitive emotional regulation have led to improved sleep quality (e.g. Gellis et al., 2013; Ong et al., 2012), including among university students (Gellis et al., 2013).

A factor that may compound the impact of time demands on students’ sleep is when students are not proactive in prioritizing their sleep (Felix et al., 2017). University students’ fail to view sleep as a priority (Coveney, 2013). In fact, only 6% of young adults state sleep is one of their most important priorities (National Sleep Foundation, 2018). In light of the numerous physical and mental health outcomes tied to sleep, it is concerning that university students place sleep so low on their list of priorities.

## Self-compassion

Due to the negative effects of inadequate sleep outlined (Buxton and Marcelli, 2010; Roberts and Duong, 2017), it is important to identify ways to help university students address those factors that may lead to inadequate sleep such as cognitive emotional regulation and prioritization of sleep. Self-compassion may be one factor that may promote sleep among students. One definition of self-compassion is that it is a way of relating to oneself with kindness and understanding in difficult times (Neff, 2003a; Neff and Germer, 2018). Neff (2003a) underlined three key components of self-compassion: self-kindness, common humanity, and mindfulness. Self-kindness involves being kind to oneself, instead of judgmental or critical (Neff, 2003a). Common humanity refers to viewing one’s personal problems or failures as part of being human (Neff, 2003a). Mindfulness is balancing one’s emotions: not overreacting nor dwelling on negative or stressful life events (Neff, 2003a). Neff and Germer (2018) have proposed two sides of self-compassion: the yin (i.e. comforting) and the yang (i.e. action-oriented) of self-compassion. Through the yin of self-compassion, university students should be in a better position to regulate their emotions, which should lead to good quality sleep. Through the yang of self-compassion, university students should take proactive steps to prioritize their sleep.

## Self-compassion and sleep quality

Two meta-analyses have examined the relationship between self-compassion and sleep (Brown et al., 2021; Butz and Stahlberg, 2020). Brown et al. (2021) found that among adults (*N* = 1830) self-compassion was negatively associated with disturbing sleep symptoms (*r* = −0.32, 95% CI [−0.36, −0.28]) with effect sizes ranging from −0.37 (Dvořáková et al., 2017) to −0.23 (Hu et al., 2018) among college students. Butz and Stahlberg (2020) found that among community adults (*N* = 959) self-compassion was positively related to subjective sleep (*r* = 0.30, 95% CI [0.24, 0.36]). Compared to a control condition, self-compassion interventions (*n* = 3) led to increases in sleep quality (Hedges’ *g* = 0.48, 95% CI [0.15, 0.82]; Butz and Stahlberg, 2020). It is evident that self-compassion is positively associated with (Brown et al., 2021; Kim et al., 2021) and leads to (Butz and Stahlberg, 2020) better sleep quality, but what is less well-known is *how* self-compassion leads to these effects.

Using the yin of self-compassion, university students may be able to achieve good quality sleep through implementing adaptive *cognitive emotional regulation*. Self-compassion has been associated with the ability to regulate emotions (Bakker et al., 2019; Inwood and Ferrari, 2018). For instance, self-compassion is associated with less unpleasant affective states when coping with stressful situations (Allen and Leary, 2010; Neff, 2004; Semenchuk et al., 2020), depression (Ferrari et al., 2019) and anxiety (Harwood and Kocovski, 2017), and is positively related to positive affect (Sirois et al., 2015a), adaptive cognitive emotional regulation (Inwood and Ferrari, 2018), and adaptive coping mechanisms (Allen and Leary, 2010).

As indicated by the temporal-affective self-regulation resource model of health behaviors (Sirois, 2015b) and previous research (Sirois et al., 2015a), self-compassion’s ability to facilitate adaptive emotional regulation leads to increased engagement in health behaviors (Sirois, 2015a), such as sleep (e.g. Hu et al., 2018). For instance, the positive relationship between self-compassion and sleep is mediated by lower levels of stress (Hu et al., 2018) and rumination (Butz and Stahlberg, 2018). Butz and Stahlberg (2018) found that self-compassion was related to (study 1) or increased (study 2 and 3) good sleep quality through its ability to decrease rumination. Research has been limited in exploring whether other cognitive emotional regulation strategies (e.g. acceptance) are relevant to better understanding the relationship between self-compassion and sleep quality.

Using the yang of self-compassion, university students may be able to achieve good quality sleep through taking proactive steps to prioritize their sleep. Theory and empirical research support this claim; individuals higher in self-compassion tend to avoid the experience of suffering and seek out what is in the best interest for their health and well-being (Neff, 2003a; Terry et al., 2013). Self-compassion has been associated with acts that reflect a proactive health focus such as setting, monitoring and adjusting health goals (Semenchuk et al., 2018; Terry and Leary, 2011), and engaging in health-promoting behaviors, including sleep (Dunne et al., 2018; Sirois et al., 2015a). No research has examined whether taking a proactive health focus may mediate the relationship between self-compassion and sleep quality.

## Study objective

The objective of this study was to examine whether, among a sample of university students, self-compassion is negatively related to poor sleep quality and whether this relationship is mediated by: (1) cognitive emotional regulation strategies and/or (2) a proactive health focus. Based on theory (Neff and Germer, 2018; Terry and Leary, 2011) and past research (e.g. Butz and Stahlberg, 2018) we hypothesize that self-compassion will be negatively related to poor sleep quality and this relationship would be mediated by engaging in cognitive emotional regulation strategies and taking a proactive health focus.

## Methods

### Participants

This study was approved by the Ethics Review board at the University of Manitoba (ethics #: HS22278) and employed a cross-sectional, online design. The sample included 193 university students who were between the ages 18 and 24 years (94%), mostly female (65.5%), Caucasian (52%), single (92.2%) and were employed part-time (46.6%), employed full time (4.1%), a full-time student (47.2%), or were out of work (2.1%). The highest level of education completed by most participants was high school (47.7%). An a priori power analysis was conducted based on recommendations from Fritz and Mackinnon (2007); 148 participants were required to obtain adequate power (0.80) to detect a small-medium effect size (0.26) for both the α and β paths (Fritz and Mackinnon, 2007). A convenience sampling strategy was employed where introductory psychology students were recruited through a participant pool at the Canadian university. This study’s only eligibility criterion was that participants needed to be a university student. Participants were granted credits toward research participation as a course requirement.

### Measures

#### Demographics

Participants began by responding to demographic questions which examined sex, ethnicity, marital status, highest level of education, and current employment status.

#### Self-compassion

Participants completed the Self-Compassion scale (Neff, 2003b) to examine self-compassion levels. This 26-item scale consists of six subscales: self-kindness, self-judgment, common humanity, isolation, mindfulness, and over-identification. Participants responded using a five-point Likert scale ranging from 1 (*almost never*) to 5 (*almost always*). Negatively worded items were reverse-scored and a grand mean was calculated for overall self-compassion. Higher scores on the self-compassion scale indicate the individual is more self-compassionate. Within the current study, this scale demonstrated high internal consistency (α = 0.88).

#### Sleep quality

Participants completed the Pittsburgh Sleep Quality Index (PSQI; Buysse et al., 1989). This 19-item measure assesses: subjective sleep quality, sleep latency, sleep duration, sleep efficiency, sleep disturbance, use of sleep medication, and daytime dysfunction (Buysse et al., 1989). Participants responded by selecting options which included “*not during the past month*,” “*less than once a week*,” “*once or twice a week*,” and “*three or more times a week.*” Items were summed and then a mean global sleep quality score was created (Buysse et al., 1989). Higher scores on the PSQI indicate worse sleep quality. Within the current study, this measure demonstrated good internal consistency (α = .75).

#### Proactive health focus

Participants completed the Proactive Health Focus scale (PHF; Terry et al., 2013). This 10-item scale is an adaptation of the Proactive Personality Scale (Bateman and Crant, 1993) that describes a proactive approach to health. A sample item is, “If I notice something about my health that I don’t like, I work to fix it.” The Proactive Health Focus scale utilizes a seven-point Likert scale ranging from 1 (*strongly agree*) to 7 (*strongly disagree*). Items were summed and then a mean proactive health focus score was created (Terry et al., 2013). Higher scores indicate the individual takes a more proactive approach to their health. Past research has indicated that this measure has strong reliability (*a* = 0.87; Bateman and Crant, 1993). Within the current study, this measure had high internal consistency (*a* = 0.88).

#### Cognitive emotional regulation

The Cognitive Emotional Regulation Questionnaire (Garnefski et al., 2001) was used to measure participants’ use of certain regulation strategies. This 36-item scale assesses nine different cognitive emotional regulation strategies consisting of: self-blame, acceptance, rumination, positive refocusing, refocus on planning, positive reappraisal, putting into perspective, catastrophizing, and other blame (Garnefski et al., 2001). This scale asks participants to respond using a five-point Likert scale ranging from 1 (*almost never*) to 5 (*almost always*). Items of each cognitive emotional regulation strategy were summed. Higher scores on each subscale indicates the individual uses the cognitive emotional regulation strategy more often. Within the current study, this measure demonstrated good internal consistency (α = 0.76).

#### Careless responding

Due to the study taking place online, there was cause for concern that careless responding would occur (Meade and Craig, 2012). To protect against this, two careless responding measures were inserted throughout the online survey.

### Procedure

Participants first completed online consent and then proceeded to answer questions related to their demographic characteristics, level of self-compassion, cognitive emotional regulation strategies, proactive health focus, and poor sleep quality through SurveyMonkey. Once all questionnaires were completed, participants were debriefed about the study objectives.

### Data analysis

Data screening and cleaning were completed according to recommendations of Tabachniak and Fidell (2007), Pallant (2010) and Field (2017). All variables had under 5% missing data; therefore missing values were estimated using mean substitution (Tabachniak and Fidell, 2007). Data from participants who failed the careless responding items were removed. Before conducting analyses all assumptions were met (Tabachniak and Fidell, 2007).

To test the hypothesis that self-compassion would negatively relate to poor sleep quality, a bivariate correlation was conducted. To test the hypothesis that cognitive emotional regulation strategies and proactive health focus would mediate the relationship between self-compassion and poor sleep quality separate mediation analyses were conducted. PROCESS version 3.5 for SPSS using 5000 bootstrap samples and a 95% confidence interval (Hayes, 2017; Model 4 was selected) were used for all analyses. To reach significance, the bootstrap interval of the indirect effect could not include zero (Hayes, 2013). For Model 1, self-compassion was entered as the independent variable, sleep quality was the outcome, and proactive health focus was the mediator. For Model 2, a parallel mediation analysis was conducted (Kane and Ashbaugh, 2017) using self-compassion as the independent variable, sleep quality as the outcome and each cognitive emotional strategy was entered in one block as the mediators.

### Data sharing statement

The current article includes the complete raw data-set collected in the study including the main de-identified data set (without imputing missing values), code book, syntax files, and log files for analyses. All the files are available in the Figshare repository and as Supplemental Material via the SAGE Journals platform. The data contains participant demographics, self-compassion, PSQI variables, proactive health focus, and cognitive emotional regulation strategies. The outputs and syntax contain information on the bivariate correlations between self-compassion, global sleep quality and all main variables, the mediation analyses, and the frequencies of all main variables. The supplemental tables include descriptive for each scale.

### Results

A total of 200 participants completed the online questionnaire. Data from seven participants were deleted due to their failure to appropriately respond to a careless responding items, leaving 193 participants to be included in the data analysis. Results indicated that self-compassion was negatively associated with poor sleep quality (*r* = −0.34, *p* < 0.001). Therefore, self-compassionate individuals had better sleep quality (as indicated by a lower score on the PSQI). A correlation table for all main variables can be found in Supplemental Table 1.

### Mediation results

#### Pathway 1

Proactive health focus did not mediate the relationship between self-compassion and poor sleep quality. Further, proactive health focus did not predict poor sleep quality (Supplemental Figure 1). The direct effect of self-compassion on sleep quality was significant, *b* = −1.87, 95% CI [−2.72, −1.01], *t* = −4.32, *p* < 0.001. A negative beta indicated that as self-compassion increased, scores on sleep quality decreased, which was hypothesized as higher scores on the PSQI indicated poorer sleep quality. Self-compassion significantly predicted proactive health focus, *b* = 0.54, 95% CI [0.30, 0.78], *t* = 4.41, *p* < 0.001.

#### Pathway 2

Self-blame mediated the relationship between self-compassion and poor sleep quality (see Supplemental Figure 2). There was a significant indirect effect of self-compassion on poor sleep quality through the cognitive emotion regulation strategy self-blame, *b* = 0.54, 95% CI [0.04, 1.04]. Further, the direct effect of self-compassion on poor sleep quality was significant, *b* = −1.99, 95% CI [−3.22, −0.77], *t* = −3.22, *p* = 0.001. This indicates that as self-compassion levels increased, levels of poor sleep quality decreased. No other cognitive emotional regulation strategies were significant (Supplemental Table 2).

## Discussion

We examined the relationship between self-compassion and poor sleep quality, and determined whether cognitive emotional regulation strategies and proactive health focus mediated this relationship. Self-compassion was negatively related to poor sleep quality. The negative relationship between self-compassion and poor sleep quality was partially mediated by self-blame. No other cognitive emotional regulation strategies were significant mediators. The relationship between self-compassion and poor sleep quality was not mediated by a proactive health focus. These results extend the self-compassion literature as no other researchers, to our knowledge, have examined proactive health focus, and its relationship to sleep. In addition, although rumination (Butz and Stahlberg, 2018) and stress (Hu et al., 2018) have been examined as mediators between self-compassion and sleep quality, we are the first to examine many other cognitive emotional regulation strategies as mediators of this relationship.

### Self-compassion and sleep quality

Self-compassion has been linked to beneficial health behaviors including healthy eating (Dunne et al., 2018) and exercising (Wong et al., 2021). Recently, researchers have uncovered a link between self-compassion and sleep quality (e.g. Brown et al., 2021). The current study contributes to this small body of research by confirming that self-compassion is negatively related to poor sleep quality.

### Cognitive emotion regulation and sleep

The results of our study indicate that self-compassion is negatively associated with poor sleep quality through its association with less self-blame. This finding suggests that the more self-compassionate students were, the less they relied on self-blame regulation strategies, which offers a possible explanation for their better sleep quality. The finding that self-blame mediated the relationship between self-compassion and sleep is consistent with theory. A central component of self-compassion is self-kindness, (Neff and Germer, 2018), a reaction that opposes self-blame. This aspect of self-kindness is crucial in the ability to treat oneself with self-compassion (Neff and Germer, 2018) and has been shown to be negatively correlated with self-blame (Phillips, 2019).

Self-compassion has been negatively associated with self-blame (Hamrick and Owens, 2019; Sirois et al., 2015b). In addition, previous research has highlighted that coping strategies such as self-blame, rumination, and catastrophizing, associate with sleep difficulties in young adults (Amaral et al., 2016, 2018). Within the current study, students who were more self-compassionate were less likely to use self-blame strategies, which was associated with better sleep. As university students may use self-blame strategies for many reasons, such as when they experience poor grades (Ding and Curtis, 2020), it is important to find factors that reduce this tendency. Due to the cross-sectional design of this study, future research should continue to examine whether teaching individuals how to be self-compassionate leads to better sleep quality through reducing self-blame.

Not all cognitive emotional regulation strategies mediated the relationship between self-compassion and poor sleep quality (e.g. acceptance, positive refocusing). However, most cognitive emotion regulation strategies were significantly related to self-compassion (with the exception of acceptance; Supplemental Table 1). It may be that although commonly related to self-compassion, certain cognitive emotional regulation strategies impact sleep more than others. For instance, among a sample of university students Latif et al. (2019) found that cognitive reappraisal was not associated with sleep quality, while expressive suppression was. Ellis et al. (2019) also found that although emotion suppression directly predicted sleep quality, cognitive reappraisal did not. Future research should examine whether cognitive emotional regulation strategies not yet examined mediate the relationship between self-compassion and sleep quality (such emotion-focused strategies; Vandekerckhove and Wang, 2018) or whether strategies that are antecedent versus response-focused (Gross, 2002) explain the relationship differently.

### Proactive health focus and sleep

Self-compassion should lead to better sleep quality because self-compassionate people are proactive with regard to their health and well-being (Neff, 2003a; Terry and Leary, 2011). In the present study self-compassion was associated with students taking a proactive health focus. This finding is also consistent with past research (e.g. Semenchuk et al., 2020). For instance, using a cross-sectional design Terry and colleagues (2013) found that self-compassionate individuals stated they would seek advice from a medical professional sooner upon experiencing symptoms than those lower in self-compassion. Getting good quality sleep is one way that students could be proactive with their health as it would help them avoid suffering in the form of drowsiness and poor health outcomes resulting from a lack of sleep (Buxton and Marcelli, 2010; Taylor and Bramoweth, 2010). However, our results indicated that taking a proactive health focus did not play a large role in how self-compassion may relate to poor sleep quality among university students. This finding is surprising given that, theoretically, self-compassion should make students prioritize their sleep through their proactive focus on health (Neff, 2003a; Terry et al., 2013).

A possible reason for this null finding is that the scale used within this study was a general measure of proactive health focus; it was not focused specifically on being proactive with sleep. It could be that self-compassionate students chose to be proactive with other health behaviors, such as physical activity or nutrition, which may have not impacted their subjective sleep quality. Research has indicated that students often have many demands and that “sleeping more and reducing other activities [is a less] desirable option” (Paterson et al., 2019: 5). A poll by the National Sleep Foundation (2018) found that 35% of university students prioritized fitness and nutrition versus the 6% who prioritized sleep. It may be that self-compassionate individuals recognize their time restraints and chose the behavior that they believe is best for their health and well-being (e.g. exercise). Participants within the current study were not asked about other health behavior engagement therefore, this is merely speculation. Future research should examine whether self-compassion leads to improved sleep quality through prioritizing sleep specifically (e.g. sleep hygiene practices).

#### Strengths and limitations

This study is the first to examine whether the relationship between self-compassion and poor sleep quality can be explained by individuals taking a proactive health approach. This study adds to previous research by examining a wide variety of cognitive emotional regulation strategies and their impact on sleep quality. There are some study limitations that should be noted. This study used a convenience sample of university students; this limits the generalizability of our study. We employed a cross-sectional design; therefore we cannot not confirm causality nor can we confirm the temporal ordering of variables (Fairchild and McDaniel, 2017). Participants were asked to recall how they typically respond when facing negative or unpleasant experiences in order to assess their cognitive emotional regulation strategies. Future research should use ecological momentary assessment methods to reduce recall bias (Colombo et al., 2020). While Neff (2016) has referred to self-compassion as healthy way of relating to oneself, she (Neff, 2003a) and others (e.g. Diedrich et al., 2014) have also referred to self-compassion as an emotion regulation strategy. These varying definitions raise questions about the extent to which emotion regulation is a component versus a correlate of self-compassion. In the future, researchers need to be clear what self-compassion is and how it is/is not distinct from other constructs. Lastly, we did not account for participants’ current mental state; research has found “at risk mental state” individuals report impaired sleep quality (Clarke et al., 2021).

## Conclusion

This study contributes to the small body of literature on the relationship between self-compassion and sleep. In a population where fewer than 1/3 get the recommended 8 hours of sleep per night (Lund et al., 2010), examining why and how students can sleep better is important. This study highlights that self-compassion may help university students achieve better sleep through its ability to reduce self-blame. Future research should continue to examine potential mechanisms of the relationship between self-compassion and poor sleep quality through longitudinal and randomized trial designs.

## Research Data

sj-pdf-1-hpq-10.1177_13591053211047148 – for Self-compassion and sleep quality: Examining the mediating role of taking a proactive health focus and cognitive emotional regulation strategies Click here for additional data file. sj-pdf-1-hpq-10.1177_13591053211047148 for Self-compassion and sleep quality: Examining the mediating role of taking a proactive health focus and cognitive emotional regulation strategies by Brittany N Semenchuk, Samantha Onchulenko and Shaelyn M Strachan in Journal of Health Psychology This article is distributed under the terms of the
Creative Commons Attribution 4.0 License ( http://www.creativecommons.org/licenses/by/4.0/)
which permits any use, reproduction and distribution of
the work without further permission provided the original
work is attributed as specified on the SAGE and Open
Access pages ( https://us.sagepub.com/en-us/nam/open-access-at-sage).

sj-pdf-11-hpq-10.1177_13591053211047148 – Supplemental material for Self-compassion and sleep quality: Examining the mediating role of taking a proactive health focus and cognitive emotional regulation strategies Click here for additional data file. Supplemental material, sj-pdf-11-hpq-10.1177_13591053211047148 for Self-compassion and sleep quality: Examining the mediating role of taking a proactive health focus and cognitive emotional regulation strategies by Brittany N Semenchuk, Samantha Onchulenko and Shaelyn M Strachan in Journal of Health Psychology

sj-pdf-12-hpq-10.1177_13591053211047148 – Supplemental material for Self-compassion and sleep quality: Examining the mediating role of taking a proactive health focus and cognitive emotional regulation strategies Click here for additional data file. Supplemental material, sj-pdf-12-hpq-10.1177_13591053211047148 for Self-compassion and sleep quality: Examining the mediating role of taking a proactive health focus and cognitive emotional regulation strategies by Brittany N Semenchuk, Samantha Onchulenko and Shaelyn M Strachan in Journal of Health Psychology

sj-pdf-3-hpq-10.1177_13591053211047148 – for Self-compassion and sleep quality: Examining the mediating role of taking a proactive health focus and cognitive emotional regulation strategies Click here for additional data file. sj-pdf-3-hpq-10.1177_13591053211047148 for Self-compassion and sleep quality: Examining the mediating role of taking a proactive health focus and cognitive emotional regulation strategies by Brittany N Semenchuk, Samantha Onchulenko and Shaelyn M Strachan in Journal of Health Psychology This article is distributed under the terms of the
Creative Commons Attribution 4.0 License ( http://www.creativecommons.org/licenses/by/4.0/)
which permits any use, reproduction and distribution of
the work without further permission provided the original
work is attributed as specified on the SAGE and Open
Access pages ( https://us.sagepub.com/en-us/nam/open-access-at-sage).

sj-sav-2-hpq-10.1177_13591053211047148 – for Self-compassion and sleep quality: Examining the mediating role of taking a proactive health focus and cognitive emotional regulation strategies Click here for additional data file. sj-sav-2-hpq-10.1177_13591053211047148 for Self-compassion and sleep quality: Examining the mediating role of taking a proactive health focus and cognitive emotional regulation strategies by Brittany N Semenchuk, Samantha Onchulenko and Shaelyn M Strachan in Journal of Health Psychology This article is distributed under the terms of the
Creative Commons Attribution 4.0 License ( http://www.creativecommons.org/licenses/by/4.0/)
which permits any use, reproduction and distribution of
the work without further permission provided the original
work is attributed as specified on the SAGE and Open
Access pages ( https://us.sagepub.com/en-us/nam/open-access-at-sage).

sj-sps-10-hpq-10.1177_13591053211047148 – for Self-compassion and sleep quality: Examining the mediating role of taking a proactive health focus and cognitive emotional regulation strategies Click here for additional data file. sj-sps-10-hpq-10.1177_13591053211047148 for Self-compassion and sleep quality: Examining the mediating role of taking a proactive health focus and cognitive emotional regulation strategies by Brittany N Semenchuk, Samantha Onchulenko and Shaelyn M Strachan in Journal of Health Psychology This article is distributed under the terms of the
Creative Commons Attribution 4.0 License ( http://www.creativecommons.org/licenses/by/4.0/)
which permits any use, reproduction and distribution of
the work without further permission provided the original
work is attributed as specified on the SAGE and Open
Access pages ( https://us.sagepub.com/en-us/nam/open-access-at-sage).

sj-sps-8-hpq-10.1177_13591053211047148 – for Self-compassion and sleep quality: Examining the mediating role of taking a proactive health focus and cognitive emotional regulation strategies Click here for additional data file. sj-sps-8-hpq-10.1177_13591053211047148 for Self-compassion and sleep quality: Examining the mediating role of taking a proactive health focus and cognitive emotional regulation strategies by Brittany N Semenchuk, Samantha Onchulenko and Shaelyn M Strachan in Journal of Health Psychology This article is distributed under the terms of the
Creative Commons Attribution 4.0 License ( http://www.creativecommons.org/licenses/by/4.0/)
which permits any use, reproduction and distribution of
the work without further permission provided the original
work is attributed as specified on the SAGE and Open
Access pages ( https://us.sagepub.com/en-us/nam/open-access-at-sage).

sj-sps-9-hpq-10.1177_13591053211047148 – for Self-compassion and sleep quality: Examining the mediating role of taking a proactive health focus and cognitive emotional regulation strategies Click here for additional data file. sj-sps-9-hpq-10.1177_13591053211047148 for Self-compassion and sleep quality: Examining the mediating role of taking a proactive health focus and cognitive emotional regulation strategies by Brittany N Semenchuk, Samantha Onchulenko and Shaelyn M Strachan in Journal of Health Psychology This article is distributed under the terms of the
Creative Commons Attribution 4.0 License ( http://www.creativecommons.org/licenses/by/4.0/)
which permits any use, reproduction and distribution of
the work without further permission provided the original
work is attributed as specified on the SAGE and Open
Access pages ( https://us.sagepub.com/en-us/nam/open-access-at-sage).

sj-spv-4-hpq-10.1177_13591053211047148 – for Self-compassion and sleep quality: Examining the mediating role of taking a proactive health focus and cognitive emotional regulation strategies Click here for additional data file. sj-spv-4-hpq-10.1177_13591053211047148 for Self-compassion and sleep quality: Examining the mediating role of taking a proactive health focus and cognitive emotional regulation strategies by Brittany N Semenchuk, Samantha Onchulenko and Shaelyn M Strachan in Journal of Health Psychology This article is distributed under the terms of the
Creative Commons Attribution 4.0 License ( http://www.creativecommons.org/licenses/by/4.0/)
which permits any use, reproduction and distribution of
the work without further permission provided the original
work is attributed as specified on the SAGE and Open
Access pages ( https://us.sagepub.com/en-us/nam/open-access-at-sage).

sj-spv-5-hpq-10.1177_13591053211047148 – for Self-compassion and sleep quality: Examining the mediating role of taking a proactive health focus and cognitive emotional regulation strategies Click here for additional data file. sj-spv-5-hpq-10.1177_13591053211047148 for Self-compassion and sleep quality: Examining the mediating role of taking a proactive health focus and cognitive emotional regulation strategies by Brittany N Semenchuk, Samantha Onchulenko and Shaelyn M Strachan in Journal of Health Psychology This article is distributed under the terms of the
Creative Commons Attribution 4.0 License ( http://www.creativecommons.org/licenses/by/4.0/)
which permits any use, reproduction and distribution of
the work without further permission provided the original
work is attributed as specified on the SAGE and Open
Access pages ( https://us.sagepub.com/en-us/nam/open-access-at-sage).

sj-spv-6-hpq-10.1177_13591053211047148 – for Self-compassion and sleep quality: Examining the mediating role of taking a proactive health focus and cognitive emotional regulation strategies Click here for additional data file. sj-spv-6-hpq-10.1177_13591053211047148 for Self-compassion and sleep quality: Examining the mediating role of taking a proactive health focus and cognitive emotional regulation strategies by Brittany N Semenchuk, Samantha Onchulenko and Shaelyn M Strachan in Journal of Health Psychology This article is distributed under the terms of the
Creative Commons Attribution 4.0 License ( http://www.creativecommons.org/licenses/by/4.0/)
which permits any use, reproduction and distribution of
the work without further permission provided the original
work is attributed as specified on the SAGE and Open
Access pages ( https://us.sagepub.com/en-us/nam/open-access-at-sage).

sj-spv-7-hpq-10.1177_13591053211047148 – for Self-compassion and sleep quality: Examining the mediating role of taking a proactive health focus and cognitive emotional regulation strategies Click here for additional data file. sj-spv-7-hpq-10.1177_13591053211047148 for Self-compassion and sleep quality: Examining the mediating role of taking a proactive health focus and cognitive emotional regulation strategies by Brittany N Semenchuk, Samantha Onchulenko and Shaelyn M Strachan in Journal of Health Psychology This article is distributed under the terms of the
Creative Commons Attribution 4.0 License ( http://www.creativecommons.org/licenses/by/4.0/)
which permits any use, reproduction and distribution of
the work without further permission provided the original
work is attributed as specified on the SAGE and Open
Access pages ( https://us.sagepub.com/en-us/nam/open-access-at-sage).
