# Supplementary material for: Self-compassion and sleep quality: Examining the mediating role of taking a proactive health focus and cognitive emotional regulation strategies
Source: J Health Psychol. 2021 Sep 20;27(10):2435–45. doi: 10.1177/13591053211047148 (PMC9434207; doi:10.1177/13591053211047148)
Supplement: sj-pdf-1-hpq-10.1177_13591053211047148 – for Self-compassion and sleep quality: Examining the mediating role of taking a proactive health focus and cognitive emotional regulation strategies [file sj-pdf-1-hpq-10.1177_13591053211047148.pdf]

Self-Compassion and Sleep Code Book

SC_1 to SC_26 = Self-Compassion Scale Question 1 to Question 26

PSQI_1 to PSQI_19 = Pittsburg Sleep Quality Index Question 1 to 19

Careless_1 = Careless responding item 1

Carless_2 = Careless responding item 2

CERQ_1 to CERQ_36 = Cognitive Emotional Regulation Questionnaire Question 1 to 36

PHF_1 to PHF_10 = Proactive Health Focus Question 1 to 10

RSC = Reverse SC scoring

PSQI_C1 to PSQI_C7 = Pittsburgh Sleep Quality Index Component 1 to Component 7

PSQI_GLOBAL_sleep = Pittsburgh Sleep Quality Index Global Sleep Score

Missing Values Information

Self-Compassion Grand Mean: Missing values (n = 7)

CERQ_Self-Blame: Missing values (n = 3)

CERQ_Acceptance: Missing values (n = 1)

CERQ_Rumination: Missing values (n = 0)

CERQ_Positive Refocusing: Missing values (n = 2)

CERQ_Refocus on Planning: Missing (n = 2)

CERQ_Positive Reappraisal: Missing (n = 1)

CERQ_Putting into perspective: Misisng (n = 2)

CERQ_Catastrophizing: Missing (n = 3)

CERQ_Other blame: Missing (n = 2)

Proactive Health focus: Missing (n = 4)

PSQI_C1 Overall Sleep Quality: Missing (n = 1)

PSQI_C2 Sleep Latency: Missing (n = 0)

PSQI_C3 Sleep Duration: Missing (n = 10)

PSQI_C4 Sleep Efficiency: Missing (n = 6)

PSQI_C5 Sleep Disturbances: Missing (n = 3)

PSQI_C6 Use of Sleeping Medication: Missing (n = 0)

PSQI_C7 Daytime Disfunction: Missing (n = 1)

PSQI_Global Sleep Score: Missing (n = 15)

Please see attached SPSS Output with all frequencies for main variables
