# Supplementary material for: Self-compassion and sleep quality: Examining the mediating role of taking a proactive health focus and cognitive emotional regulation strategies
Source: J Health Psychol. 2021 Sep 20;27(10):2435–45. doi: 10.1177/13591053211047148 (PMC9434207; doi:10.1177/13591053211047148)
Supplement: sj-pdf-11-hpq-10.1177_13591053211047148 – Supplemental material for Self-compassion and sleep quality: Examining the mediating role of taking a proactive health focus and cognitive emotional regulation strategies [file sj-pdf-11-hpq-10.1177_13591053211047148.pdf]

Supplemental Table 1

Relationships among main study variables and scale descriptives

Variables	Mean	Standard deviation	Range	Scale Range	Self-compassion	Sleep quality
Self-compassion	3.06	.50	1.75 to 4.53	1.00 to 5.00	1	-.34**
Sleep quality	7.20	.43	0.00 to 17.10	0.00 to 21.00	-.34**	1
Proactive health focus	5.08	.89	2.10 to 7.00	1.00 to 7.00	.30**	-.20**
Self-blame	2.60	.76	1.00 to 5.00	1.00 to 5.00	-.45**	.08
Rumination	2.88	.76	1.00 to 5.00	1.00 to 5.00	-.35**	.16*
Acceptance	3.05	.74	1.25 to 5.00	1.00 to 5.00	-.02	.11
Positive refocusing	2.53	.80	1.00 to 4.50	1.00 to 5.00	.32**	-.19**
Refocus on planning	3.12	.83	1.25 to 5.00	1.00 to 5.00	.33**	.15*
Positive reappraisal	3.15	.87	1.25 to 5.00	1.00 to 5.00	.46**	-.20
Putting into perspective	2.94	.82	1.25 to 5.00	1.00 to 5.00	.32**	-.10
Catastrophizing	2.26	.79	1.00 to 4.76	1.00 to 5.00	-.35**	.17*
Other blame	2.07	.64	1.00 to 4.26	1.00 to 5.00	-.19**	-.02

Note. N = 193; *$p < .05$; **$p < .001$

Supplemental Table 2

Indirect effects between self-compassion, cognitive emotional regulation strategies and sleep quality

CERQ Subscale	Beta	95% CI	*Bootstrapped SE*
Self-blame	.54	[.04, 1.04]	.26
Rumination	-.15	[-.56, .24]	.20
Acceptance	-.02	[-.21, .14]	.08
Positive refocusing	-.21	[-.60, .10]	.17
Refocus on planning	.08	[-.39, .61]	.25
Positive Reappraisal	-.23	[-.90, .45]	.35
Putting into perspective	.03	[-.32, .38]	.17
Catastrophizing	-.27	[-.61, .09]	.18
Other-blame	.15	[-.02, .41]	.11

Note. CERQ = Cognitive Emotion Regulation Questionnaire; CI = confidence interval; SE = standard error.

Supplemental Figure 1

Mediation Model with Self-Compassion, Proactive Health Focus, and Sleep Quality

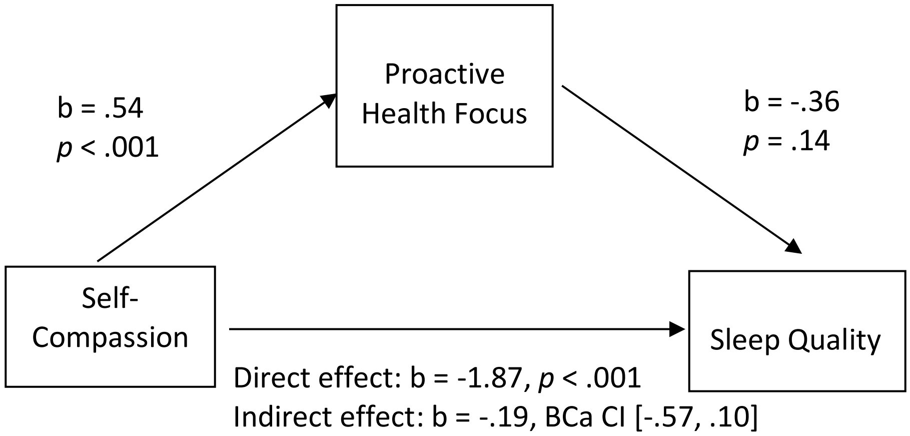

Note. CI = 95% Confidence Interval

Supplemental Figure 2

Mediation Model Between Self-Compassion, Self-Blame and Sleep Quality

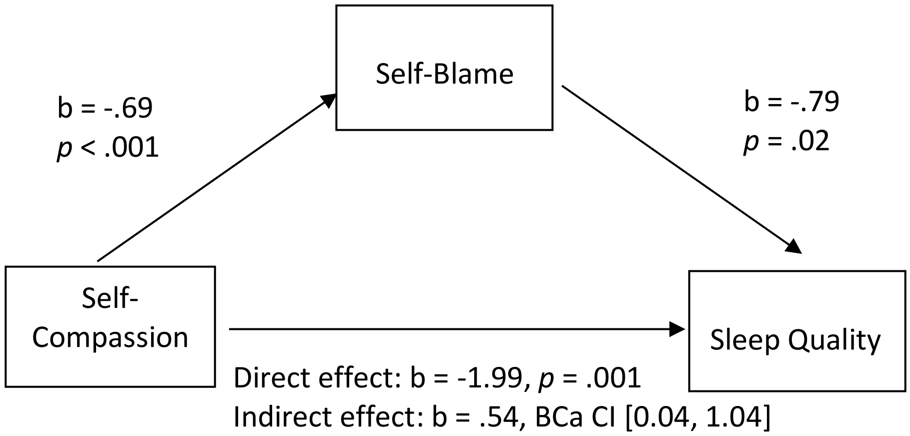

Note. CI = 95% Confidence Interval
