# Supplementary material for: Self-compassion and sleep quality: Examining the mediating role of taking a proactive health focus and cognitive emotional regulation strategies
Source: J Health Psychol. 2021 Sep 20;27(10):2435–45. doi: 10.1177/13591053211047148 (PMC9434207; doi:10.1177/13591053211047148)
Supplement: sj-pdf-3-hpq-10.1177_13591053211047148 – for Self-compassion and sleep quality: Examining the mediating role of taking a proactive health focus and cognitive emotional regulation strategies [file sj-pdf-3-hpq-10.1177_13591053211047148.pdf]

Explanatory Memo

The enclosed files include the manuscript with figures, data set, SPSS results outputs & syntax, and supplemental tables from the present study.

The data is stored on SPSS version 27 and contains participant demographic information, self-compassion, proactive health focus, cognitive emotional regulation strategies and the Pittsburg Sleep Quality Index. For easy review and understanding we have also created a codebook with the labels and missing value information.

The software used for the analysis in the present study was IBM SPSS Statistics 27.0. The attached SPSS results outputs contain the results from the Bivariate Pearson correlations and the mediation analyses that were conducted in the present study. The outputs include the results of the bivariate correlations between participants' total self-compassion scores and global sleep quality, each cognitive emotional regulation strategy, and proactive health focus. In addition, bivariate correlations are included regarding the relationship between global sleep quality, each cognitive emotional regulation strategy and proactive health focus. These results were obtained by opening SPSS, clicking Analyze > Correlate > Bivariate. Next, you select the two variables you wish to correlate and move them into the correlation box. Finally select Pearson and two-tailed and click OK. The results are also presented in Supplemental Table 1.

In addition, the outputs include the results of the mediation analyses conducted using Hayes PROCESS Macro version 3.5. Specifically, the first mediation analysis was conducted using self-compassion as the 'X' variable, sleep quality as the 'Y' variable, and proactive health focus was entered as the mediator. The second mediation analysis was conducted using self-compassion as the 'X' variable, sleep quality as the 'Y' variable, and all the cognitive emotional regulation strategies as the mediators. These results were obtained by opening SPSS, clicking Analyze > Regression > PROCESS 3.5 by Andrew Hayes > variables were entered as described above > Model 4 was chosen > Options > ensure the following are checked off: "show total effect model", "effect size". Finally click OK. These results are also presented in Figures 1 and 2 within the manuscript.

The supplemental tables for the present study include the information on the descriptive statistics, bivariate correlations, and indirect effects of all main variables.
